# Protocol for using single-cell sequencing to study the heterogeneity of NF1 nerve sheath tumors from clinical biospecimens

**DOI:** 10.1016/j.xpro.2023.102297

**Published:** 2023-05-10

**Authors:** Xiyuan Zhang, Vishaka Gopalan, Neeraja Syed, Sridhar Hannenhalli, Jack F. Shern

**Affiliations:** 1 Pediatric Oncology Branch, Center for Cancer Research, National Cancer Institute, National Institutes of Health, Bethesda, MD 20892, USA; 2 Cancer Data Science Laboratory, Center for Cancer Research, National Cancer Institute, National Institutes of Health, Bethesda, MD 20892, USA; 3 Pediatric Oncology Branch Childhood Cancer Data Initiative, Frederick National Laboratory for Cancer Research, National Cancer Institute, National Institutes of Health, Bethesda, MD 20892, USA

**Keywords:** Bioinformatics, Single Cell, Cancer, Genetics, RNAseq

## Abstract

Single-cell sequencing is a powerful technology to understand the heterogeneity of clinical biospecimens. Here, we present a protocol for obtaining single-cell suspension from neurofibromatosis type 1-associated nerve sheath tumors for transcriptomic profiling on the 10x platform. We describe steps for clinical sample collection, generation of single-cell suspension, and cell capture and sequencing. We then detail methods for integrative analysis, developmental Schwann cell trajectory building using bioinformatic tools, and comparative analysis. This protocol can be adapted for single-cell sequencing using mouse nerve tumors.

For complete details on the use and execution of this protocol, please refer to Zhang et al. (2022).^1^

## Highlights

• Procedure for single-cell suspension preparation from patient NF1 nerve sheath tumors
• Single-cell RNA sequencing using the 10x Chromium workflow
• Integrative analysis of data collected from multiple patients to correct batch effect
• Building a developmental Schwann cell trajectory for comparative analysis

## Abstract (editor highlights)

Publisher’s note: Undertaking any experimental protocol requires adherence to local institutional guidelines for laboratory safety and ethics.

## Graphical abstract

**Figure:**
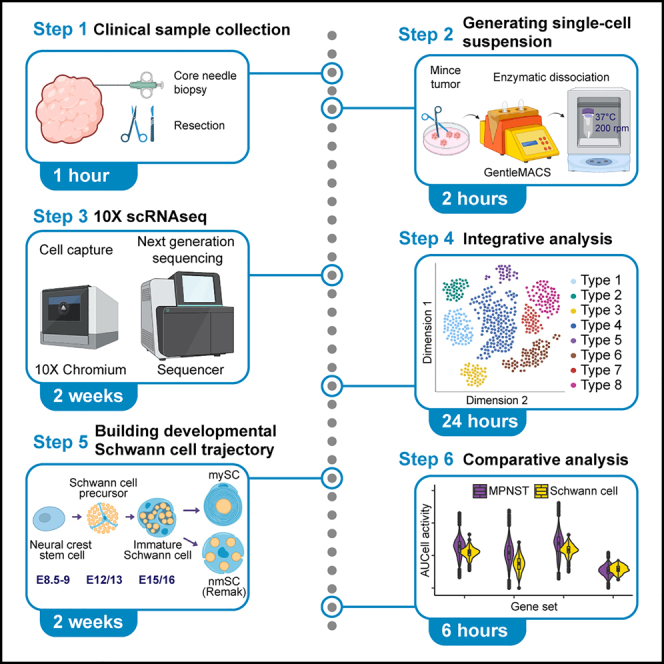

## Teaser

Single-cell sequencing is a powerful technology to understand the heterogeneity of clinical biospecimens. Here, we present a protocol for obtaining single-cell suspension from neurofibromatosis type 1-associated nerve sheath tumors for transcriptomic profiling on the 10× platform. We describe steps for clinical sample collection, generation of single-cell suspension, and cell capture and sequencing. We then detail integrative analysis, developmental Schwann cell trajectory building using bioinformatic tools, and comparative analysis. This protocol can be adapted for single-cell sequencing using mouse nerve tumors.

## Before you begin

The protocol below describes the specific steps for single-cell RNA sequencing of a single-cell suspension obtained from fresh NF1-associated nerve sheath tumors. Transcriptomic profiling of all cell types on a single-cell level is a powerful tool to understand the intra-tumoral heterogeneity of these tumors. This protocol can be used on both fresh surgical specimens as well as patient-derived xenografts to profile the transcriptome on a single-cell level.^2^

### Institutional permissions (if applicable)

Institutional permission for obtaining and studying clinical specimens should be obtained. Informed consent needs to be obtained from individual patients enrolled in a relevant clinical protocol. Informed consent was obtained on NCI IRB approved protocol (NCT01109394; IRB identifier 10C0086) for the tumor samples prospectively collected and included in this work. Samples are ordered and tracked through a clinical trial data management system. Upon receipt, the samples are assigned a unique barcode ID that is delinked from all identifying information and used for all downstream analysis.

### Preparation of tumor dissociation media on the day before the surgery

**Timing: 1 h**

1. Prepare the DNase I solution as described in the materials and equipment section.
  a. Prepare the 10× DNase I buffer.
  b. Dilute the 10× DNase I buffer with Hanks' Balanced Salt Solution (HBSS) to 1× DNase I buffer.
  c. Inject 20 mL of the 1× DNase I buffer to reconstitute the lyophilized DNase I so that the final concentration is 100 Kunitz units/mL.
  d. Store the DNase I solution in 200 μL aliquots at −80°C. They are stable for a year.
2. Prepare the sterile tumor dissociation media as described in the materials and equipment section.
  a. Weigh the dispase II and collagenase I to be dissolved in Dulbecco’s modified eagle medium (DMEM) supplemented with 10% fetal bovine serum and penicillin-streptomycin-glutamine.
  b. Filter the enzyme cocktail with a 0.22 μm filter unit and store at 4°C.

### Preparation on the day of surgery

**Timing: 1 h**

3. Warm up the sterile tumor dissociation media to 37°C.
4. Add DNase I to the sterile tumor dissociation media (100 μL per 10 mL).
5. Prepare the following items to be used in the laminar flow hood before heading to the procurement room to collect specimens.
  a. One 10 mL syringe.
  b. gentleMACS™ c-tube
  c. Surgery scalpel and sterile tweezer.
  d. 60 mm tissue culture dish.
  e. 40 μm cell strainer.

**CRITICAL:** A minimum of 10 mL of complete tumor dissociation media is needed to process each specimen. Scale up the preparation of complete tumor dissociation media and tools for each additional specimen that is scheduled to be collected and processed.

## Key resources table

**Table:** 

REAGENT or RESOURCE	SOURCE	IDENTIFIER
**Chemicals, peptides, and recombinant proteins**		

Dulbecco’s Modified Eagle Medium	Thermo Fisher	Cat# 11995081
Fetal bovine serum	Bio-Techne	Cat# S11550
Penicillin-streptomycin-glutamine (100×)	Thermo Fisher	Cat# 10378016
Collagenase I	STEMCELL Technologies	Cat# 07416
Dispase II	MilliporeSigma	Cat# D4693-1G
DNase I	Invitrogen	Cat# 18047019
Bovine serum albumin	Sigma-Aldrich	Cat# A9418
ACK lysing buffer	Thermo Fisher	Cat# A1049201
AO/PI viability dye	Nexcelom	Cat# CS2-0106-5ML
Dulbecco’s phosphate buffered saline	Sigma-Aldrich	Cat# D8537-500ML

**Critical commercial assays**		

Dead cell removal kit	Miltenyi Biotec	Cat# 130-090-101
EasySep Human CD56 Positive Selection Kit II	STEMCELL Technologies	Cat# 17855

**Deposited data**		

E9.5 neural crest cell single-cell sequencing data	Soldatov et al.^3^	GSE129114
E12.5/E13.5 adrenal medulla cell single-cell sequencing data	Furlan et al.^4^	GSE150150
Mature Schwann cell single-cell sequencing data	Wolbert et al.^5^	GSE142541
Human NF1 nerve tumor single-cell sequencing data	Zhang et al. 1	GSE183309

**Software and algorithms**		

Euler algorithm in TFBSTools	Tan and Lenhard ^6^	https://bioconductor.org/packages/release/bioc/html/TFBSTools.html
Cell Ranger v6.0.0	10× Genomics	https://support.10xgenomics.com/single-cell-gene-expression/software/downloads/latest
Seurat v4.0	Butler et al.^7^; Stuart et al.^8^	https://satijalab.org/seurat/
inferCNV	inferCNV of the Trinity CTAT Project	https://github.com/broadinstitute/inferCNV
HOCOMOCO v11	Kulakovskiy et al.^9^	https://hocomoco11.autosome.org
JASPER 2020	Fornes et al.^10^	https://jaspar.genereg.net
SCENIC	Aibar et al.^11^	https://scenic.aertslab.org
Monocle3	Cao et al.^12^ ; Trapnell et al.^13^	https://cole-trapnell-lab.github.io/monocle3/

**Other**		

0.22 μm filter unit	Millipore Sigma	Cat# SLGP033RS
10 mL Syringe	BD	Cat# 302995
MACS C-tube	Miltenyi Biotec	Cat# 130-096-334
Sterile surgery scalpel	N/A	N/A
40 μm cell strainer	Corning/Falcon	Cat# 352340
6 cm tissue culture dish	Corning	Cat# 353001
GentleMACS dissociator	Miltenyi Biotec	N/A
Cellometer Auto 2000	Nexcelom	N/A
Chromium Controller	10× Genomics	N/A
Illumina NextSeq machine	Illumina	N/A
Cell Counting Chamber	Nexcelom	Cat# SD100-514

## Materials and equipment

**Table:** Tumor Dissociation Media

Reagent	Final concentration	Amount
Dulbecco’s modified eagle medium	N/A	45 mL
Fetal bovine serum	10%	5 mL
penicillin-streptomycin-glutamine (100×)	1×	500 μL
Dispase II	1 mg/mL	50 mg
Collagenase I	1 mg/mL	50 mg
DNase I solution (100 unit/mL)	1 Kunitz unit/mL	500 μL
**Total**	**N/A**	**50 mL**

***Note:*** Prepare the Tumor Dissociation Media without adding the DNase I solution the day before the surgery. Store the sterile incomplete Tumor Dissociation Media at 4°C for a maximum of 24 h.

***Note:*** Complete the preparation of the Tumor Dissociation Media by adding the DNase I solution immediately before the surgery.

**Table:** 10× DNase I buffer

Reagent	Final concentration	Amount
Tris hydrochloride (1 M), pH 8.0	400 mM	4 mL
CaCl_2_ (1 M)	10 mM	100 μL
MgSO_4_ (1 M)	100 mM	1 mL
Hanks' Balanced Salt Solution	N/A	4.9 mL
**Total**	**N/A**	**10 mL**

***Note:*** Store 10× DNase I buffer at 18°C–25°C for a maximum of 24 months.

**Table:** DNase I solution

Reagent	Final concentration	Amount
10× DNase I buffer	1×	2 mL
Hanks' Balanced Salt Solution	N/A	18 mL
DNase I (lyophilized)	100 Kunitz unit/mL	1 vial
**Total**	**N/A**	**20 mL**

***Note:*** Store the DNase I solution in single-use aliquots for a maximum of 12 months.

### Tissue dissociator

The gentleMACS™ from Miltenyi Biotec has been utilized in this protocol. This equipment is a benchtop instrument for the semi-automated dissociation of tissues into single-cell suspensions.

***Alternatives:*** If a gentleMACS™ dissociator is not available in the laboratory, use of a 10 mL serological pipette to mix and agitate the minced tumor with Tumor Dissociation Media is acceptable.

## Step-by-step method details

### Clinical sample collection

**Timing: 1 h**

This step describes the procedure of collecting clinical samples for single-cell sequencing.

**Figure 1:**
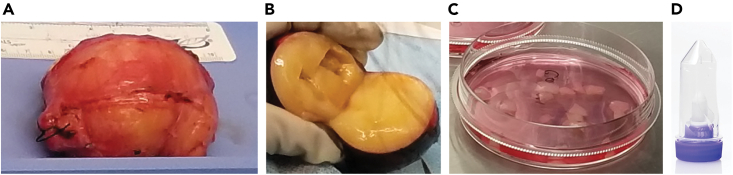
Illustration of tissue processing to achieve single-cell suspension from fresh nerve sheath tumor (A) Example NF1-associated nerve sheath tumor obtained from the operation room. (B) Picture of an inked and transected tumor with a 0.5 x 0.5 x 0.5 cm^3^ cube of tissue acquired from the tumor. (C) Picture of minced tumor with the presence of Tissue Dissociation Media in a petri dish. (D) Picture of a c-tube that can be used on a gentleMACS^TM^ dissociator. This tube can be directly placed in the incubator for shaking and enzymatic dissociation.
  b. If the specimen is a core needle biopsy obtained for diagnostic purposes.
    i. Obtain the ultrasound-guided biopsy from the area-of-interest by proceduralists in the Intervention Radiology department.
    ii. Once a core needle biopsy is obtained, immediately place the biospecimen in DMEM in a 15 mL conical tube.

1. Institutional approval and informed consent need to be in place before the usage of any clinical samples. In this protocol, the collected specimen can be obtained by surgical resection or core needle biopsy of the neurofibroma.
2. Biospecimen collection from patient neurofibroma.
  a. Resected tumor may be obtained from a debulking surgery.
    i. The surgically removed tumor comes from the operation room in a sterile container.
    ii. Open the sterile container a laminar flow hood within the procurement suite.
    iii. Apply pathology ink to the biospecimen to better visualize tumor margins.
    iv. Measure the three-dimensional size of the inked specimen with a ruler and record the measurements.
    v. Cut open the biospecimen lengthwise in the middle.
    vi. Remove one 0.5 × 0.5 × 0.5 cm^3^ cube of tissue from one side and immediately place in DMEM in a 15 mL conical tube (Figure 1)

***Optional:*** Multi-regional sampling may be necessary if there is grossly visible heterogeneity (regional coloring or texture of the resected tumor) or magnetic resonance imaging (MRI) indicated that significant heterogeneity exists within the same tumor. Process the samples from different regions of the same tumor separately in the following steps.

**CRITICAL:** It is important to keep appropriate labeling and annotation for each specimen throughout the procedure. Pictures and annotations should be properly documented.

### Generating single-cell suspensions of nerve sheath tumor

**Timing: 2 h**

This step describes how to use enzymatic dissociation to generate a single-cell suspension of nerve sheath tumors.

**Figure 2:**
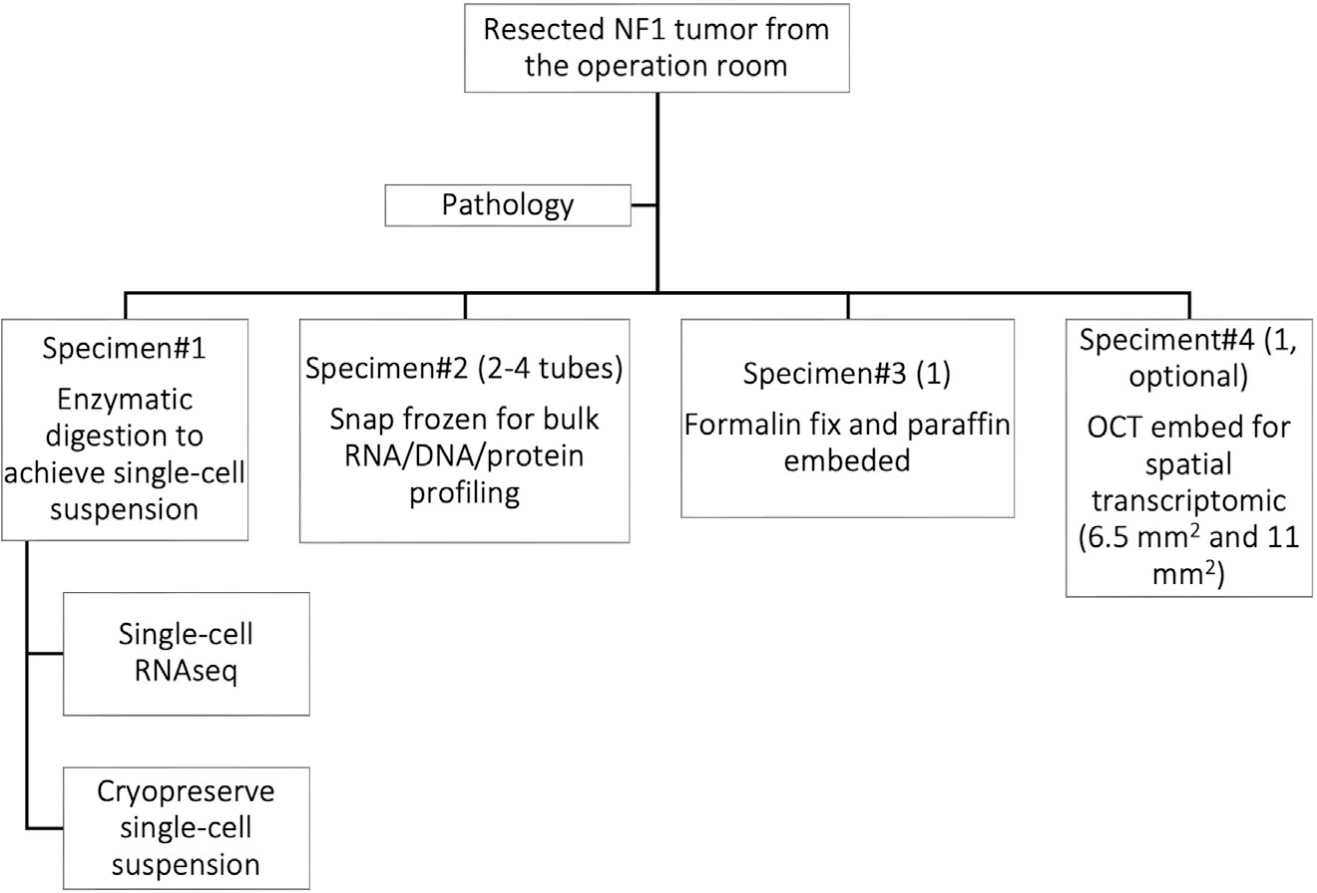
Schematic of the workflow of NF1-associated nerve sheath tumor processing from the surgical suite to the laboratory

**Figure 3:**
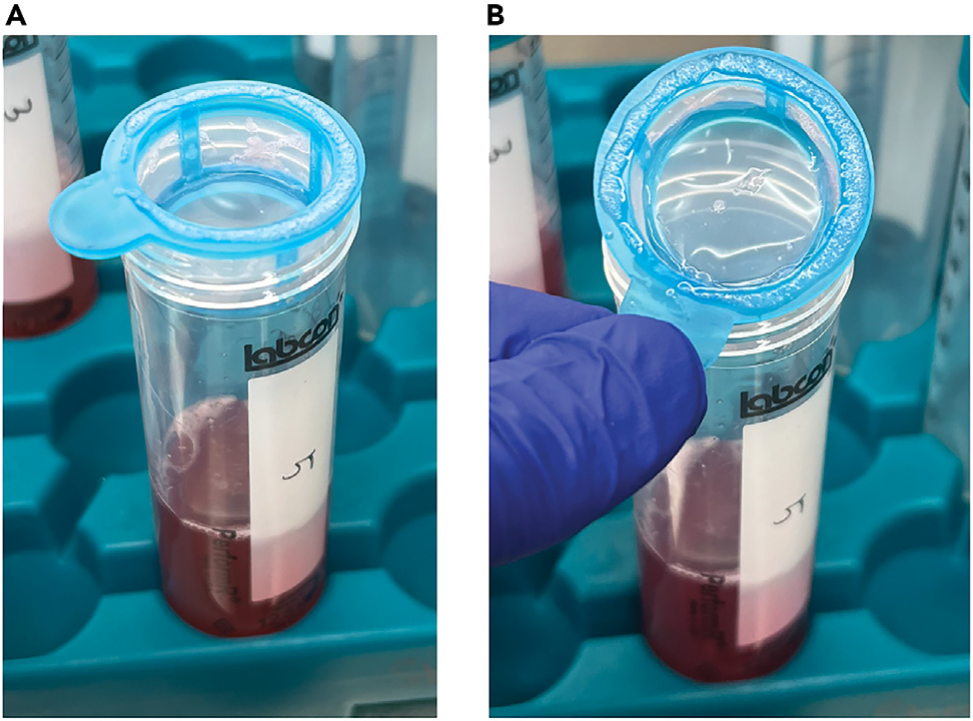
Illustration of using cell strainer to ensure the generation of single-cell suspension (A) Picture of the setup of a 40 μm cell strainer on an opened 50 mL conical tube. (B) After using a syringe plunger to mechanically force the remaining tissue chunks through the cell strainer, there should be minimal residual of tissue on the cell strainer.
  k. Add 60 μL of sterile 0.5 M EDTA solution into the filter-through single-cell suspension and mix by inverting the tube.
  l. Centrifuge the cells at 300 *g* for 5 min to collect the cell pellet.
    **CRITICAL:** Always add EDTA to the single-cell suspension to a final concentration of 1 mM before spinning the cells to prevent aggregation.
  m. Using a 10 mL serological pipette, carefully remove all but 0.5 mL of the media. Resuspend the cell pellet.
  n. Count the cells using Acridine Orange/Propidium Iodide (AO/PI) fluorescent dye by mixing 20 μL cell suspension with 20 μL dye (Nexcelom) and loading 20 μL of the mixture to the disposable slide (Nexcelom).
  o. Insert the slide into the chamber of a fluorescent cell counter.
    ***Note:*** Cellometer Auto 2000 was used in this study but other automatic cell counter or manual counting using a hemocytometer can also be used.
    ***Note:*** Live cells fluoresce green fluorescent and dead cells fluoresce orange fluorescent.
    ***Note:*** The automatic cell counter counts the number of green fluorescent cells as live cells and the number of orange fluorescent cells as dead cells.
  p. Calculate the cell concentration in the single-cell suspension using a dilution factor of 2.
  q. Wash the cells with 20 mL of DMEM and repeat steps l-p.
  r. Determine the cell concentration and viability again using AO/PI dye and a fluorescent cell counter (Figure 4).

3. Dissection of the biospecimen and curation of resected tumor
  a. Spray the outside of the conical tube with 70% ethanol and open it under a laminar flow hood. The tissue will be processed in the hood.
  b. Transfer the biospecimen to a 60 mm cell culture plate. Using sterile surgical scalpel and tweezers, dissect the tumor into pieces of 2 × 2 × 2 mm^3^ (Figure 2)
    i. Fix one piece of the biospecimen in 10% buffered formalin solution at 25°C for a maximum of 24 h before transferring to 80% alcohol.
      ***Note:*** Process this piece for the paraffin embedded tissue block. This piece can be used for downstream validation experiments.
    ii. Place two to three pieces individually in Eppendorf tubes and snap freeze in liquid nitrogen.
      ***Note:*** Store these pieces at −80°C. They may be used for bulk sequencing or other experiments.
    iii. Mince the remaining tumor into 0.5 × 0.5 × 0.5 mm^3^ pieces in the cell culture plate.
      **CRITICAL:** When processing a core needle biopsy, no dissection or curation is needed. Directly transfer the biopsy biospecimen into a c-tube for enzymatic dissociation.

4. Enzymatic dissociation
  a. For each sample to be dissociated, prepare 10 mL of complete Tumor Dissociation Media.
  b. Use a 10 mL serological pipette to add the Tumor Dissociation Media to the minced tumor and transfer them to a c-tube (Miltenyi).
  c. Place the c-tube on the gentleMACS™ dissociator and run “hTumor_2.1” twice.
  d. Shake the c-tube at 37°C for up to 40 min at 200 rpm.
    **CRITICAL:** To process a core need biopsy sample, reduce the incubation time for enzymatic dissociation in step 4d to 20 min.
  e. Place the c-tube on the gentleMACS™ dissocator and run “hTumor_3.1” twice.
  f. Place a 40 μm cell strainer on a 50 mL conical tube under the laminar flow hood.
  g. Use a 10 mL serological pipette to agitate the dissociated sample in the tube under the hood to agitate the tumor into single-cell suspension.
  h. Transfer the suspension in the c-tube (dissociated sample may contain small pieces of tumor chunk) onto the cell strainer.
  i. Use the plunger from a 10 mL syringe to push the tumor chunks on the cell strainer.
  j. Rinse the cell strainer with 10 mL DMEM twice. The filter-through will contain ∼ 30 mL of dissociated tumor cells (Figure 3).

**Figure 4:**
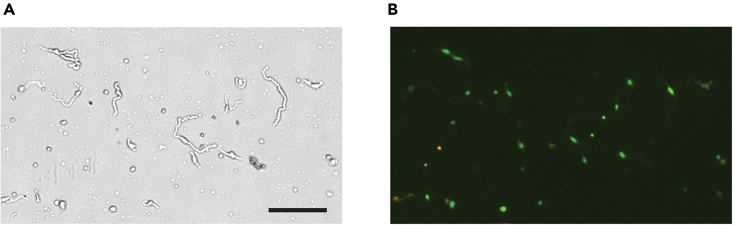
Example of cell count and viability determined by AO/PI staining on a fluorescent cell counter (A) Bright field image. Note that the “string”-shaped cells are neoplastic Schwann cells isolated from a NF1-associated nerve sheath tumor. (B) Fluorescent image showing live nucleated cells stained by Acridine Orange (green) and dead nucleated cells stained with Propidium Iodide (orange). Scale bar = 200 μm.
  s. Adjust the cell concentration to be in the range of 700–1200 live cells per microliter in DMEM before proceeding to the single-cell sequencing.
  t. Process the cells immediately for single-cell capture.
    **CRITICAL:** The success of the downstream single-cell RNA sequencing depends on good preparation of single-cell suspension with the viability above 70% and devoid of cell debris and dead cells.
    ***Optional:*** If a gentleMACS™ dissociator is not available in the lab, use a 10 mL serological pipette to mix and agitate the minced tumor in Tumor Dissociation Media.
    ***Optional:*** If significant amount of red blood cells exists in the sample, which will appear pink in the cell pellet, add 10 mL ACK lysing buffer to the resuspended cells after step 4m and incubate the sample at room temperature for 5 minutes, followed by PBS wash twice.
    ***Optional:*** If the cell viability is less than 60%, an optional dead cell removal step maybe added after step 4p. Commercially available dead cell removal kit (Miltenyi Biotec) has been used and proved to be successful in enrichment of live cells. Be cautious that up to 50% of total cell loss should be expected using the dead cell removal kit.
    ***Optional:*** It is possible to increase the content of Schwann cells in the dissociated single-cell suspension without affecting the downstream single-cell sequencing. We have previously identified that CD56 (encoded by *NCAM1*) is a unique cell surface marker expressed by mouse^14^ and human^1^ Schwann cells. Using a CD56 antibody-based selection kit (STEMCELL), it is possible to enrich for Schwann cells isolated from the tumor for downstream single-cell sequencing.

### Single-cell RNA sequencing using the 10x platform

**Timing: 1–2 weeks**

This step describes the procedure for 3′ gene expression profiling of single cells including single-cell capture using 10x chromium platform, library preparation and sequencing. The recommended user guide “CG000315_ChromiumNextGEMSingleCell3-_GeneExpression_v3.1_DualIndex__RevE.pdf” is followed.

5. Single-cell capture.
  a. Count the cells again using fluorescent automated cell counter to determine viability and concentration before loading the cells on to a NextGEM chip for single-cell capture.
  b. If necessary, adjust the cell concentration to be in the range of 700–1200 live cells/μL by diluting or concentrating the cells from previous step.
  c. Count the cells one more time to check the cell concentration and viability just before loading.
  d. Load the cells with the target recovery of 6000 cells per capture lane.

***Note:*** For most tumors processed in this project, two capture lanes per tumor sample are used, targeting 12,000 cells per tumor sample.

**CRITICAL:** Make sure not to introduce air bubbles while loading the cell suspension and other reagents into the wells.

**CRITICAL:** Before loading the cells for capture, make sure to check the viability and concentration of the samples one more time.

6. Library preparation.
  a. After the GEM generation, check the consistency and volume of the GEMs.
    ***Note:*** If the quality of the GEMs is not as expected, for example (1) low volume of GEMs is observed, (2) GEMs appear less cloudy, or (3) transparent liquid is observed instead of the cloudy GEMs, it indicates either a wetting failure or partial emulsification has occurred.
    **CRITICAL:** It is necessary to repeat the capture if the quality of the GEMs is not as expected. Before loading the cells for recapture, make sure to completely resuspend and count the cells again.
    **CRITICAL:** After GEM formation, pay close attention to the volume, opacity, and consistency of the GEM for a clog or possible wetting failures.
  b. Proceed to GEM-RT incubations immediately after GEM generation.
    **Safe pause point**: store the tubes at 4°C for up to 72 h or at −20°C for up to a week.
  c. Process the GEMs through GEM recovery, cleanup with Dynabeads, and prepare the cDNA.
    **Safe pause point**: store the cDNA at 4°C for up to 72 h or at −20°C for up to 4 weeks.
  d. Check the quality and concentration of cDNA using one of the following quantification methods: Bioanalyzer, TapeStation, or Labchip.
    ***Note:*** The quality and concentration of the cDNA provide some insights about the progress of the assay and helps for troubleshooting later if needed.
  e. Continue with final library preparation from cDNA that includes fragmentation, end repair and A-tailing, adaptor ligation, size selection using SPRIselect beads and sample index PCR.
    **Safe pause point**: store the final library at 4°C for up to 72 h.
  f. After the completion of sample index PCR, perform one additional round of size selection using SPRIselect beads.
    **Safe pause point:** store the tubes at 4°C for up to 72 h or at −20°C for long-term storage.
  g. Check the quality and concentration of the final library with a Bioanalyzer, TapeStation, or Labchip.
    **CRITICAL:** Pay close attention to the size distribution and concentration of the final library and the size of the adaptor dimer peak.

7. Sequencing.
  a. The Final 3′ gene expression dual indexed libraries are standard Illumina paired end constructs.
    ***Note:*** Read 1 is 28 base pare (bp) long which encodes 16 bp 10x cell barcode and 12 bp unique molecule index (UMI). Read 2 is 90 bp long which contains the cDNA fragment that is later aligned to the reference genome to indicate the expressed RNA. The sample index sequences, i5 and i7, are 10 bp long each. They allow the pooling of several 10x cDNA libraries for sequencing.
  b. Calculate the molarity of the libraries using the concentration and the size of the final library.
  c. Prepare 2 nM library for each cDNA library, pool the 2 nM libraries from multiple 10x cDNA libraries and re-check the molarity.
  d. Dilute the pooled libraries to 1 nM as the final loading concentration.
    ***Note:*** The final loading concentration is dependent on the type of Illumina sequencer used, and it needs to be optimized for each sequencer.
  e. Sequence the pooled libraries using an Illumina sequencer.
  f. When sequencing multiplexed 10x libraries, perform an initial shallow sequencing run to normalize the libraries so that each library will be sequenced at a similar molarity.
    **CRITICAL:** The flow cell to use for running the libraries depend on the number of multiplexed libraries and the number of cells targeted. For example, for five multiplexed libraries and each with a target of approximately 6000 cells, the sequencing depth will be: 50,000 reads × 6000 cells × 5 libraries = 1.5 billion reads. NovaSeq S2 flow cell will yield approximately 1.3–1.6 billion single-end reads.
    **Pause point:** According to the manufacturer’s protocol, safe pause points are available after the fresh single-cell suspension has been captured at steps 6b, 6c, 6e, and 6f.
    **CRITICAL:** The time to complete this step varies based on the number of samples. The more samples captured and to be sequenced, the longer it will take to complete this step. One to two weeks are sufficient to process a typical 2 lanes of single-cell capture from one tumor sample.

### Integrative data analysis, marker genes identification, and cell type annotation

**Timing: 24 h**

This step describes how to perform basic integrative analysis, marker gene identification and cell type annotations of single-cell RNAseq data generated from NF1 nerve sheath tumors.

8. Use the standard 10x Genomics cellranger (version 6.0.0) pipeline to extract fastq files and to perform data processing.
  a. Demultiplex the raw base call files (BCL) generated by the Illumina sequencer into fastq files using cellranger mkfastq.
  b. Run cellranger count pipeline for alignment to the reference genome (refdata-gex-GRCh38-2020-A), filtering, barcode and UMI counting.
9. Apply SoupX^7^ to the count matrix of each capture lane to correct the ambient RNA contamination in the data.
  a. Load the count matrix generated from cellranger.
  b. Manually specify the contamination fraction to 10%, 20%, 30%, and 40%.
  c. Generate corrected count matrix to be used in downstream analysis.
  d. Visually examine the expression of known markers of each cell type generated from using each contamination fraction. For example, B cell marker CD79B should be expressed in B cells but not the other cell types.
  e. Once a desired contamination fraction is determined, use this contamination fraction to generate the corrected count matrix to be used in the next step.

10. Apply Scrublet^8^ to the SoupX-corrected read count matrix for the prediction of doublets in the data.
  a. Based on the number of cells captured per capture lane, a predicted doublet rate is calculated as the following:
    $$Predicted\;doublet\;rate\;\left(\%\right)=\frac{number\;of\;cells\;captured}{10000}\times 0.8$$
  b. Use the calculated predicted doublet rate on a per capture lane basis in function scr.Scrublet in Scrublet. This step will define the expected_doublet_rate.
  c. Remove marked doublets before advancing to the next step in this data analysis pipeline.

11. Use Seurat^15^ for the integrative analysis of data from multiple captures to correct for potential batch effect.
  a. Remove cells with low quality (nFeature_RNA < 500, nFeature_RNA > 6000, or percent of mitochondrial gene fraction > 20%).
  b. Normalize corrected gene counts using the “LogNormalize” method with the scale.factor set to 10000.
  c. Calculate the top 3000 most variable genes using the method “vst”.
  d. Assign the cell cycle identity of each cell using the function CellCycleScoring.
  e. Identify anchors using the FindIntegrationAnchors function with the setting “dims = 1:40”.
  f. Apply anchor-based reciprocal principal component analysis (rPCA)^16^ to integrate data from different capture lanes.
  g. Scale the data with the unwanted source of variations regressed out ("percent.mt", "nCount_RNA", "nFeature_RNA", "S.Score", "G2M.Score").
  h. Utilize the standard dimensional reduction for the clustering analysis using the RunPCA (npcs = 40), RunUMAP (reduction = “pca”, dims = 1:40), FindNeighbors (reduction = “pca”, dims = 1:40), and FindClusters (resolution = 0.8) functions in Seurat.
  i. Identify cluster biomarkers by finding the differentially expressed genes in each cluster with comparison to all other clusters, using the FindAllMarkers function in Seurat with the setting “only.pos = TRUE, min.pct = 0.25, logfc.threshold = 0.25” to derive the most highly expressed marker genes of each cluster.
12. Manually annotated cell types based on the identified marker genes in each cluster.

***Optional:*** Several other R-based or Python-based tools are available for integrative analysis of single-cell RNA sequencing data. Users may choose any suitable bioinformatic tools for these steps.

***Optional:*** Instead of manually specifying a contamination fraction, the function autoEstCont can be used in SoupX to automatically identify a desired contamination fraction (rho).

### Building a normal Schwann cell developmental trajectory

**Timing: 2 weeks**

This step describes how to build a normal Schwann cell developmental transcriptional trajectory using publicly available single-cell RNAseq data. All source code used in this step can be found on: https://github.com/vishakagopalan/nf_hackathon.

13. Data download and processing.
  a. Identify publicly available single-cell RNAseq data obtained from mouse normal developing Schwann cells. Download the read count matrices of the following data from GEO database.
    i. Obtain data of neural crest stem cells at embryonic day 9.5 (E9.5) from GSE129114.
    ii. Obtain data of Schwann cell precursors at E12.5 and E13.5 from GSE150150.
    iii. Obtain data of mature myelinating and non-myelinating Schwann cells from adult mice from GSE142541.
      ***Optional:*** Emerging single-cell RNAseq datasets of neural crest stem cells, Schwann cell precursors, as well as other developing and differentiated Schwann cell subtypes are available to be integrated. These datasets can also be used to build the normal Schwann cell developmental trajectory.
  b. Use published cell-type annotations for GSE129114 (Table S9 in source publication) and GSE142451.
  c. For GSE150150, perform batch correction and clustering using the default parameters in Seurat, and identify annotated cell types based on marker genes employed in the source publication.
  d. Merge all datasets together in Seurat.
  e. Use the library size (sequencing depth in each cell), cell cycle status, and batch as co-variates to estimate the p-value of differential expression for each gene.

14. Trajectory analysis in Monocle3.
  a. Down-sampling of reads from datasets that contain a much larger library sizes is required.
    ***Note:*** Among the three datasets that we use in this study, GSE129114 and GSE150150 are downsampled to match the library size distribution of cells in GSE142541.
  b. Use Monocle3 to construct a trajectory from the merged data.
    i. Merge the three datasets after the down-sampling.
    ii. Perform batch correction using the internal function of Monocle3.
    iii. Use E9.5 neural tube cells as the root state of the trajectory (these cells will be assigned a pseudotime value of 0) to compute pseudotime values of the remaining cells.

15. Regulon analysis in SCENIC.
  a. Regulons are defined as a target gene set for each transcription factor, active in each developing cell type, based on the presence of binding sites within 10 kilobases of the transcription starting site of expressed genes.
  b. Retain transcription factors whose mean regulon activity is higher than 0.03 in at least one of the cell types for future analysis.
16. Determine markers of developing Schwann cells using the FindAllMarkers function in Seurat.
17. Compute cell cycle status (G2M, S, or G1 phase scores) using AddModuleScore.

***Note:*** The cell cycle status of each cell is considered as co-variates for determining the p-values of the log-fold changes of each differentially expressed gene.

### Comparative analysis between neoplastic Schwann cell and malignant peripheral nerve sheath tumor cell

**Timing: 6 h**

This step describes how to transcriptionally project the neoplastic Schwann cells onto the normal Schwann cell developmental trajectory. All source code used in this step can be found on: https://github.com/vishakagopalan/nf_hackathon.

18. Compute at most 300 signature genes of each cell type.

***Note:*** Signature genes of the developing Schwann cells are markers of each cell type from step 16 that have an adjusted p-value of less than 0.1.

19. Comparison between Schwann cells and MPNST malignant cells.
  a. Derive marker genes of neoplastic Schwann cells and MPNST malignant cells from the integrative analysis of patient nerve sheath tumors in step 12. Specifically, using the function FindMarkers with the setting (only.pos = TRUE, min.pct = 0.25, logfc.threshold = 0.25), derive the most highly expressed marker genes in both malignant and Schwann cells.
  b. As part of the function of SCENIC package, score the activity of each of the signature gene sets (signature genes derived from step 15) in each cell using the AUCell function.
  c. Compare the AUCell activities of each developmental Schwann cell types between malignant MPNST cells and Schwann cells from the patient MPNST single-cell dataset using two-sided Wilcoxon test.

## Expected outcomes

This protocol is intended for the identification of intra-tumoral heterogeneity of nerve sheath tumors using single-cell RNA sequencing on the 10x Genomics platform. Depending on the type of specimen, approximately 50,000 (one core needle biopsy) – 1,000,000 (2 × 2 × 2 mm^3^ tissue from a resected tumor) live cells are expected to be obtained from one patient nerve sheath tumor. Cell viability is determined with AO/PI staining on a fluorescent automatic cell counter, ranging from 70%–95% (Figure 4). Typically, two lanes of single-cell suspension are captured from each tumor, aiming to recover 12,000 live cells.

Using the Tissue Dissociation Buffer coupled with shaking at 37°C has been an efficient method to dissociate fresh nerve sheath tumors. With the help of an automatic dissociator (gentleMACS™ from Miltenyi Biotec) before and after the incubation, minced tumor pieces are adequately mixed with the Tissue Dissociation Buffer. If an automatic dissociator is not available, agitation with a 10 mL serological pipette can be used to mix the sample. Minimal residual tissue chunks should be observed after the dissociation when the sample is filtered through a 40 μm cell strainer (Figure 2B). Mechanical force of the residual tissue through the cell strainer using a syringe plunger followed by rinsing the strainer with DMEM is helpful to increase the yield without any compromise of cell viability.

Schwann cells are considered the cell-of-origin of NF1-associated nerve sheath tumors. The content of Schwann cells ranges between 1%–80% in the dissociated single-cell suspension. We have previously identified that CD56 (encoded by *NCAM1*) is a unique cell surface marker of mouse^14^ and human^1^ Schwann cells. Using a CD56 antibody-based selection kit, we have previously enriched for CD56-positive human Schwann cells in an atypical neurofibroma, followed by successful single-cell sequencing on a 10x platform. This method increased the Schwann cell content from 30% to over 90% in the tumor and was proved not to affect the downstream single-cell sequencing.

Single-cell sequencing on the 10x Genomics platform has been adapted in this protocol. Using the commercial kit and following the manufacturer’s protocol will generate reliable and reproducible single-cell sequencing data.

This protocol provides a general guideline for single-cell RNAseq data analysis as presented in Zhang et al.^1^ To correct potential batch effects that is caused by captures, samples, cell cycle status of the cell, as well as other variables, anchor-based integration^16^ should be performed when merging sequencing data from multiple biospecimens, followed by dimension reduction and cell clustering. The cell numbers of the NF1 tumors included in the study^1^ at each of the quality control steps are listed in Table 1. The integrated data is processed in the downstream analysis so that the shared cell populations (cell types) can be directly compared between pathological modalities. Patient-specific or capture-specific cell populations should not dominate the discovery of intratumoral heterogeneity within these tumor datasets.

**Table 1:** Cell numbers of the NF1 tumor captured and presented in this study at each stage of the quality control

	Initial cell number	Cell number post Scrublet	Cell number post filtering
Primary MPNST	27,784	25,832	20,469
Metastatic MPNST	47,442	44,537	35,803

## Limitations

This protocol is intended to process fresh nerve sheath tumors that are directly obtained from the surgical suite. Timely acquisition and processing of the biospecimen are required to preserve the transcriptome and maintain viability of the cells. Typically, a tumor is processed within 15 min after it is acquired, and the enzymatic dissociation should not last more than 2 h. We do realize that a timely procedure may not be available given the unforeseen outcome of a complicated surgery. While not optimal, to best preserve the freshness of biospecimens when dissociation is not immediately feasible, placing the freshly obtained sample in the MACS™ tissue storage solution (Miltenyi Biotec) at 4°C can maintain the cell viability for up to 48 h. If needed, single-nuclei RNA sequencing can also be employed in frozen tumor samples. However, the data integration and compatibility will need to be further explored when handling both single-cell and single-nuclei RNA sequencing data are used.

Based on previous experience and given the tissue composition of nerve sheath tumors, the recipe of enzymatic cocktail is composed of dispase II, collagenase I, and DNase I. However, this recipe maybe highly tissue-specific and may not work efficiently for other tissue types to generate single-cell suspension. To use this protocol in other tissue types, optimization of the enzyme cocktail and time for incubation time is necessary to ensure the yield and cell viability.

In order to build a normal Schwann cell developmental trajectory, we used publicly available single-cell sequencing data from mouse embryos (for neural crest stem cell subtypes and Schwann cell precursors), newborns (for immature Schwann cells), and adult mice (for differentiated Schwann cells). Mouse gene symbols were converted to the available human equivalent before this integrated dataset was used inform the difference between neoplastic Schwann cells and malignant cells. However, there are known discrepancies of gene function in mouse versus human during the development. Future work integrating developing human Schwann cells from the different developmental stages is warranted to better understand the de-differentiated phenotype that was observed in human MPNST cells.

Finally, a useful addition to these analyses would be the inclusion of Schwann cells from normal nerve. However, human normal nerve from the same patient (sites where this is no neurofibroma) or healthy donors (normal *NF1* status genetically) are typically spared in clinical debulking surgeries. On the other hand, single-cell RNAseq of mouse NF1-associated nerve tumors often contain cell clusters of neurons.^14^ The different approaches used in human and mouse sampling should be taken into considerations when discrepancies are observed.

## Troubleshooting

### Problem 1

The viability of cells determined by the fluorescence cell counter is lower than 70% (step 4).

### Potential solution

Cell viability will decrease as time passes after the surgical resection. To reduce cell loss and death, the procedures of sample acquisition, dissection, and enzymatic dissociation need to be completed within 1–2 h after the tumor comes out of the operation room. If live cells are surrounded by cell debris, additional cell washes with DMEM may help clean up the cell debris. If a large quantity of dead cells (orange) determined by the fluorescence cell count exist, a step of dead cell removal using a magnetic separator (Miltenyi Biotec) may be employed. Note that any additional dead cell removal steps will cause significant cell loss (∼50%). It is not recommended to perform dead cell removal if limited number of cells are available.

### Problem 2

Large quantity of red blood cells exists in the sample with the cell pellet appearing pink (step 4).

### Potential solution

It is possible that red blood cells exist in large quantity in a single-cell suspension, especially when the sample is obtained as a core needle biopsy. As determined by a fluorescent cell counter, red blood cells appear in the bright field image as small round cell-like particles, but they are not stained with AO/PI dye since they do not have nuclei. Presence of these cells will significantly hamper the result and it is critical to sufficiently remove them if the cell pellet appear pink or red after step 4l. It is recommended to use 10 mL ACK lysis buffer to resuspend the bloody cell pellet, followed by 5 min incubation at room temperature and two times of washes. Cell concentration and viability need to be determined again before proceeding to the subsequent steps.

## Resource availability

### Lead contact

Further information and requests for resources and reagents should be directed to and will be fulfilled by the lead contact, Jack F. Shern (john.shern@nih.gov).

### Materials availability

This study did not generate new unique reagents.

## Data Availability

This study did not generate a new dataset. The single-cell RNA sequencing dataset of the human neurofibromatosis type 1 (NF1) malignant nerve sheath tumors mentioned in this protocol was previously described by Zhang et al. (2022)*.*
